# A Modular Vision System for Practical Object Detection on Resource-Constrained Humanoid Robots

**DOI:** 10.3390/biomimetics11060363

**Published:** 2026-05-22

**Authors:** Meng Cheng Lau, Nicolas Pottier

**Affiliations:** School of Engineering and Computer Science, Laurentian University, Greater Sudbury, ON P3E 2C6, Canada; npottier@laurentian.ca

**Keywords:** humanoidrobotics, object detection, YOLOv9, ROS, embedded AI, real-time vision

## Abstract

Deploying modern deep learning-based vision systems on humanoid robots remains challenging due to limited onboard computational resources and legacy software constraints. This paper presents a modular vision system for practical object detection on resource-constrained humanoid platforms, based on the YOLOv9 framework. The proposed architecture adopts a dual-environment design, decoupling the perception pipeline from the robot control system to enable compatibility between modern deep learning libraries and a ROS-based platform. To support efficient deployment, task-specific lightweight models are trained and integrated into a modular pipeline optimized for CPU-only inference. The system is evaluated across multiple task scenarios derived from the FIRA RoboWorld Cup (Hurocup) competition, including Marathon, Basketball, and Archery. Performance is assessed in terms of detection accuracy and computational efficiency, demonstrating that reliable perception can be achieved at 4–8 FPS under constrained hardware conditions. The results show that the proposed approach improves robustness compared to traditional geometric vision methods, particularly in dynamic and visually complex environments, while maintaining practical responsive task-level perception for robotic decision-making. The work highlights the trade-offs between accuracy, computational cost, and system responsiveness and demonstrates the feasibility of deploying modern object detection models on embedded humanoid platforms.

## 1. Introduction

Recent advances in deep learning have significantly improved the performance of computer vision systems, particularly in object detection. Models such as those in the YOLO (You Only Look Once) family [1] achieve strong performance in both accuracy and speed, enabling real-time perception in domains such as autonomous driving, surveillance, and robotics. However, their deployment in humanoid robotic systems remains limited.

A primary challenge is the constrained computational environment of many humanoid platforms. These systems often operate on CPU-only hardware without dedicated GPUs, making real-time inference difficult. In addition, legacy robotic software frameworks such as Robot Operating System (ROS) introduce compatibility constraints with modern deep learning libraries, further complicating integration. As a result, many robotic systems still rely on traditional geometric vision methods, which are computationally efficient but lack robustness in complex environments.

To address these challenges, this work proposes a modular vision system designed for resource-constrained humanoid robots. The system integrates a YOLOv9-based perception pipeline within a dual-environment architecture, enabling modern deep learning models to operate alongside a ROS-based control system. Communication between environments is achieved through a WebSocket-based bridge. This design choice prioritizes software compatibility and modular integration rather than communication latency optimization.

The system design is inspired by principles observed in biological vision systems, particularly the modular organization of perception processes. Rather than relying on a single unified model, the proposed approach employs task-specific lightweight models tailored to individual robotic tasks. This reflects the specialization of perceptual pathways and enables efficient use of limited computational resources.

The system is evaluated in the context of FIRA Hurocup tasks, including Marathon, Basketball, and Archery, which require diverse perception capabilities under varying conditions. Performance is assessed in terms of detection accuracy and computational efficiency, with a focus on practical deployment.

The main contributions of this work are: (1) A modular vision architecture enabling integration of modern deep learning models with legacy robotic systems. (2) A task-specific modeling strategy for efficient deployment on CPU-constrained platforms. (3) An empirical evaluation demonstrating that moderate frame rates (4–8 FPS) can still support operational robotic perception under constrained computational conditions. (4) A comparative analysis between deep learning-based and traditional geometric vision approaches.

## 2. Background

This section provides an overview of the key concepts and technologies relevant to this work. We first review traditional and deep learning-based approaches to object detection, followed by an overview of the YOLO framework. We then discuss the role of vision modules in robotic systems and highlight the challenges associated with deploying modern perception models on resource-constrained humanoid robots. Finally, we identify the research gap addressed by this study.

### 2.1. Object Detection

Computer vision encompasses a wide range of subfields, with object detection playing a critical role in practical robotic applications such as autonomous vehicles [2], agriculture [3,4,5], surveillance [6,7], and healthcare [8,9]. Modern object detection systems must both classify objects and localize them using bounding boxes, enabling fast and reliable decision-making in dynamic environments.

Early object detection methods relied on handcrafted feature descriptors. Techniques such as Histogram of Oriented Gradients (HOG) demonstrated strong performance in pedestrian detection [10] and facial recognition [11]. Scale-Invariant Feature Transform (SIFT) improved robustness through keypoint detection [12,13], while Speeded-Up Robust Features (SURF) further enhanced computational efficiency for real-time tasks [14,15]. Although effective in controlled settings, these approaches often struggle with complex scenes and varying environmental conditions.

The emergence of deep learning, particularly convolutional neural networks (CNNs), fundamentally transformed object detection. Two-stage detectors such as Fast R-CNN prioritize accuracy and have been successfully applied in domains such as infrastructure inspection and facial analysis [16,17,18]. In contrast, one-stage detectors, including SSD and the YOLO family, emphasize efficiency by directly predicting object classes and bounding boxes in a single forward pass, making them suitable for practical robotic applications.

Despite these advances, the adoption of modern object detection techniques in humanoid robotics remains limited. Existing research in humanoid systems often focuses on interaction, control, and learning [19,20,21,22], with comparatively less emphasis on robust perception. This gap highlights the need for efficient and deployable vision systems tailored to humanoid platforms.

Recent advances in embedded artificial intelligence (AI) have further emphasized the importance of deploying deep learning models on resource-constrained platforms. Lightweight architectures such as MobileNet [23,24] and EfficientDet [25], along with inference optimization frameworks such as TensorRT [26], have been widely adopted to balance detection accuracy and computational efficiency in real-time systems [27,28,29]. These developments highlight the importance of reducing model complexity, optimizing inference pipelines, and leveraging hardware-aware acceleration techniques to meet strict power and memory constraints.

However, many existing approaches assume access to GPU acceleration or specialized hardware. In contrast, this work considers deployment on CPU-only humanoid platforms, where such resources are not available. Instead of relying solely on extremely lightweight architectures with reduced representational capacity, this work adopts a YOLO-based approach to achieve a practical balance between detection accuracy and computational efficiency under constrained conditions.

This motivates the use of efficient one-stage detection frameworks, such as YOLO, which are discussed in the following section.

### 2.2. YOLO: You Only Look Once

The YOLO (You Only Look Once) framework is a widely used one-stage object detection approach known for its balance between speed and accuracy [30,31]. Unlike two-stage detectors, YOLO processes the entire image in a single pass, enabling real-time performance suitable for embedded and robotic applications.

YOLOv1, introduced in 2015, achieved 45 FPS with 63.4% average precision (AP) [32]. Subsequent versions introduced significant improvements in both accuracy and efficiency. YOLOv2 improved detection performance and robustness [33], while YOLOv3 enhanced multi-scale detection capabilities [34]. Later versions, including YOLOv4 through YOLOv7, introduced architectural and training optimizations such as mosaic augmentation and advanced feature aggregation [35,36,37].

One of the most recent iterations, YOLOv9, incorporates innovations such as programmable gradient information and the GELAN architecture, further improving efficiency and detection performance [1]. While YOLO has been extensively applied in domains such as autonomous driving and surveillance, its deployment on resource-constrained humanoid robots remains limited.

### 2.3. Robotic Applications & Vision Modules

Vision modules play a critical role in robotic systems by serving as the interface between perception and decision-making. They enable robots to interpret visual data for tasks such as navigation, object manipulation, and interaction with dynamic environments. Unlike standalone object detection systems, robotic vision modules must operate within a closed-loop control framework, where perception outputs directly influence robot actions in real time.

In practice, designing vision modules for robotic platforms involves additional constraints beyond detection accuracy. These include limited computational resources, strict real-time requirements, and the need for robust performance under varying environmental conditions. Many robotic systems, particularly humanoid platforms, operate without dedicated GPU acceleration, making efficient inference a key challenge.

Traditional vision approaches, such as color segmentation and template matching [38,39], are computationally efficient but often lack robustness in complex environments. In contrast, deep learning-based methods provide improved generalization but introduce higher computational demands. As a result, deploying modern object detection models in robotics requires careful system-level design to balance accuracy, efficiency, and responsiveness. In many practical robotic systems, moderate processing rates may still be sufficient when perception is used for high-level task guidance rather than low-level closed-loop motor control.

Furthermore, robotic vision systems must handle challenges such as motion-induced blur, changing viewpoints, partial occlusion, and dynamic lighting conditions. These factors can significantly impact detection reliability and, consequently, task performance. Therefore, the development of effective vision modules for humanoid robots requires not only strong detection capability but also practical considerations of integration, latency, and real-time operation within the control loop.

### 2.4. Research Gap

Despite significant advances in deep learning-based object detection, its deployment in humanoid robotics remains limited due to computational constraints and integration challenges. Existing robotic vision systems often rely on task-specific configurations or traditional methods that lack robustness in dynamic and unstructured environments.

In particular, there is a lack of generalized vision frameworks capable of operating efficiently on resource-constrained humanoid platforms while maintaining robustness across multiple tasks. This limitation becomes especially evident in scenarios such as FIRA Hurocup competitions, where robots must perform diverse, visually demanding tasks under varying environmental conditions.

To address this gap, this work proposes a modular, YOLO-based vision system designed for CPU-only humanoid robots. Inspired by efficient biological perception systems, the approach emphasizes adaptability, modularity, and practical performance. This design is loosely inspired by the separation of perceptual processing pathways in biological vision systems, where different streams specialize in distinct tasks such as object recognition and spatial awareness. By deploying recent advances in lightweight object detection and system-level optimization, this work aims to enable the practical deployment of deep learning-based vision in humanoid robotics. This work demonstrates that modern deep learning-based perception can be practically integrated into CPU-constrained humanoid robotic systems through careful system-level design and optimization.

## 3. Methodology

This section describes the design and implementation of the proposed vision system. We first introduce the robotic platform and hardware configuration, followed by the preparation and training of the YOLOv9 models. We then present the system integration within ROS, including the communication architecture between components. Finally, we outline the experimental setup and evaluation methodology used to assess system performance.

### 3.1. Hardware Configuration

**Figure 1:**
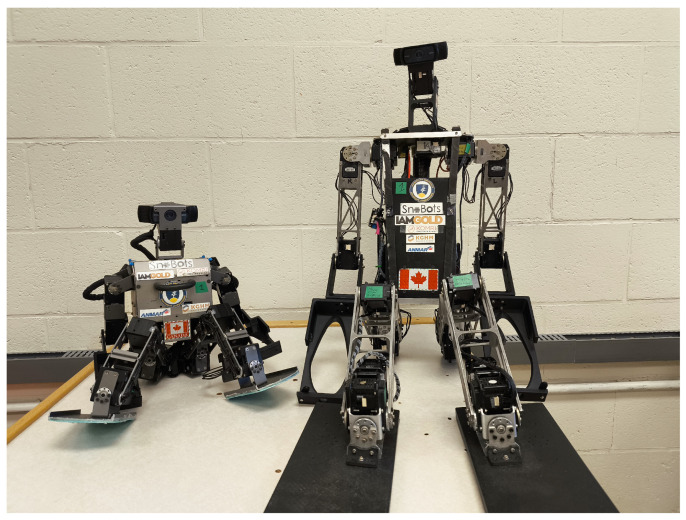
LIMRL’s two humanoid robots. ROBOTIS-OP3, also known as Oscar (pictured **left**), will be used for this implementation. In the future, the vision module will also be installed on Polaris (pictured **right**).

The humanoid platform used in this work is the ROBOTIS-OP3 (Figure 1), a 51 cm tall, 3.5 kg robot equipped with 20 XM430-W350-R actuators and a Logitech C920 HD Pro webcam. The ROBOTIS-OP3 humanoid platform has been used in a variety of interactive and task-oriented robotic applications, including synchronized musical performances and autonomous entertainment-oriented interactions [40,41]. The system is powered by an 11.1 V 1800 mAh LiPo battery or an external power supply. Minor modifications include 3D-printed hands and feet to support task execution. The OP3 onboard computer is equipped with a 6th Generation Intel Core i3-6100U (2.3 GHz) and 8GB DDR4 SODIMMs 2133 MHz. These specifications highlight the computational constraints under which the system operates.

The OP3 operates on ROS Kinetic, enabling reliable hardware control and modular software integration. Due to its limited onboard computational resources, all perception tasks must be optimized for CPU-based execution. Local image processing is used to reduce latency, while the system architecture supports optional offloading to external computing resources when required. Communication with the robot is established via Ethernet or VNC-based remote access.

### 3.2. YOLOv9 Preparation and Training

The objective of this work is to integrate the YOLOv9 object detection framework into a humanoid robotic platform to enable responsive task-level perception under limited computational resources. The software environment is designed to support efficient communication between the vision module and the robot’s control system, combining pre-trained deep learning models, open-source tools, libraries, and middleware to ensure flexibility and reproducibility.

The implementation is developed within ROS, which provides a modular framework for integrating the camera system, the YOLO inference pipeline, and the robot’s actuators into a unified architecture. The system is implemented primarily in Python 3.12, leveraging PyTorch 2.5 for model training and inference and OpenCV for image processing.

YOLOv9 is selected due to its strong balance between detection accuracy and computational efficiency. Architectural improvements such as Programmable Gradient Information (PGI) enhance gradient flow and weight optimization, while the GELAN structure improves both training and inference performance. These characteristics make YOLOv9 suitable for deployment in resource-constrained robotic systems.

To evaluate the system in realistic scenarios, models are trained for three FIRA Hurocup events: Archery, Marathon, and Basketball. Each task requires detecting distinct objects and supporting different robot behaviors. Table 1 summarizes the objects included in the training datasets for each event. For the archery task, the model detects a moving target and estimates its center for aiming. In the marathon task, the robot follows a red guiding line and classifies directional arrow markers (left, right, and forward), which may appear under varying orientations. For the Basketball event, the robot detects a ball, performs a pick-up motion, identifies the basket, and executes a throwing action once within an appropriate distance.

To maintain responsive performance on CPU-only hardware, multiple lightweight models are trained independently for each task rather than using a single unified model. This task-specific approach reduces model complexity and improves inference efficiency.

The training process begins with the collection and preprocessing of task-specific datasets. Each dataset contains approximately 150 images per class, representing a balance between dataset size and training efficiency under practical data collection constraints. Images are annotated using bounding boxes in Roboflow’s YOLOv9 format, where each annotation includes a class identifier followed by normalized bounding box coordinates. Roboflow is also used to perform data augmentation, including rotation, brightness variation, scaling, and other transformations to improve model generalization under varying environmental conditions [42,43].

The models are trained using the PyTorch framework on an NVIDIA RTX 1650 GPU. Training is conducted for 100 epochs with a batch size of six and an input resolution of 640 × 640 pixels, initialized from pre-trained MSCOCO weights [44]. A close mosaic augmentation threshold of 50 is applied to enhance data diversity.

To mitigate the risk of overfitting, early stopping is implemented with a patience value of five epochs, meaning training is halted if no improvement is observed within five consecutive epochs. This value is determined empirically through experimentation with higher patience values (20, 15, and 10), which were found to increase the risk of overfitting without improving performance. Early stopping with lower patience values has been widely used to reduce the risk of overfitting in deep learning training pipelines, particularly for smaller datasets [45].

Three lightweight model architectures are evaluated: YOLOv9-Tiny (YOLOv9-T), YOLOv9-Small (YOLOv9-S), and YOLOv9-Medium (YOLOv9-M). These variants are selected due to their lower computational requirements, making them suitable for deployment on CPU-only robotic platforms. All models are trained on an external workstation via command-line execution to reduce training time and avoid additional computational load on the robot.

### 3.3. ROS Integration

The proposed vision module is tightly integrated within the Robot Operating System (ROS) to enable practical communication between the camera, the inference pipeline, and the OP3’s actuators. The system adopts a modular node-based architecture consisting of three primary components: the YOLO inference node, a listener node, and event-specific control nodes.

ROS operates using a publisher–subscriber communication model, where nodes exchange data asynchronously through named topics. This design improves modularity and scalability, as nodes do not require direct knowledge of each other. In the proposed system, the YOLO node publishes detection results, which are received and reformatted by the listener node before being distributed to event-specific modules.

A key challenge arises from compatibility constraints: the OP3 platform relies on ROS Kinetic with Python 2.7 and OpenCV 3.3.1, whereas YOLOv9 requires Python 3 and modern deep learning libraries. To address this, the YOLO inference node is implemented in a Python 3.12 virtual environment using PyTorch and OpenCV 4.11, isolated from the ROS environment.

The YOLO node subscribes to the camera topic, processes incoming frames resized to the model’s input resolution, and performs object detection. Detection outputs, including bounding boxes, class labels, and confidence scores, are generated in real time. To improve computational efficiency, inference can be limited to approximately 20 inference updates per second instead of processing every frame of the input stream. The detection results are transmitted via WebSocket as JSON messages, allowing the YOLO node to operate independently of ROS constraints while still maintaining communication with the system.

**Figure 2:**
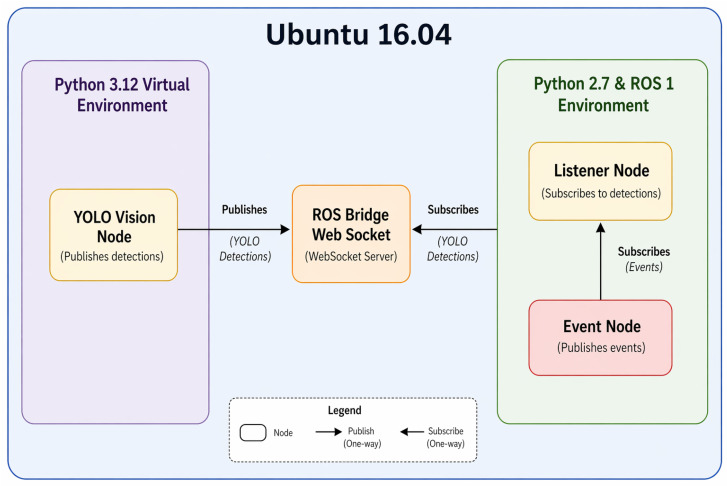
Flowchart illustrating the interaction between ROS nodes and the dual-environment architecture. Different colors indicate separate environments and functional modules. The ROSBridge WebSocket enables communication between the Python 3-based YOLO inference node and the ROS-based control system.

As shown in Figure 2, a listener node is introduced to bridge the communication between the YOLO inference environment and ROS. This node receives detection data via WebSocket and republishes it to ROS topics (e.g., /yolo/detections) in a compatible format. This design decouples the perception module from the control system, improving maintainability and enabling independent updates to the vision pipeline.

The WebSocket bridge was primarily introduced to address software compatibility constraints between legacy ROS Kinetic and modern deep learning frameworks requiring Python 3.x support, rather than as a latency-optimized communication mechanism. Prior studies have reported that JSON serialization overhead in ROSBridge-based systems is typically small relative to neural network inference and overall system-level latency, particularly when transmitting lightweight semantic data such as bounding boxes and class labels [46,47].

Event-specific nodes subscribe to the detection topics and use the received data to identify target objects and execute corresponding actions, such as navigation, tracking, or object interaction. High-level behavior and object tracking are handled through custom Python logic, while low-level actuator control is managed using the Dynamixel SDK.

Due to the limited computational capability of the OP3’s onboard CPU, several optimization strategies are applied to achieve practical performance. Training is performed externally on a dedicated workstation, while onboard inference is optimized through frame skipping and input resizing to 640 × 640 pixels. Additionally, the trained YOLO model is converted from PyTorch to ONNX format and subsequently to OpenVINO, which provides optimized CPU inference. Quantization is applied during this process to further reduce computational overhead. The modular architecture of ROS allows the system to scale efficiently, enabling alternative detection models to be integrated with minimal changes to the overall system.

### 3.4. Performance Evaluation

The performance of the proposed vision module is evaluated through a combination of controlled laboratory experiments and task-based assessments aligned with FIRA Hurocup scenarios. The evaluation focuses on three key aspects: detection accuracy, computational efficiency, and system responsiveness. The objective of this evaluation is not to establish strict real-time guarantees or characterize low-level control latency, but rather to assess whether modern deep learning-based perception can provide sufficiently responsive guidance on CPU-constrained humanoid platforms.

Experiments are first conducted in a controlled indoor environment with consistent lighting and minimal background interference. This setup allows for reproducible measurement of baseline metrics such as detection accuracy and frame processing rate. The system is subsequently evaluated in dynamic task scenarios, including Marathon, Basketball, and Archery, where environmental variability such as object motion, orientation changes, and partial occlusion are present.

Detection performance is quantified using standard object detection metrics, including precision, recall, mean Average Precision at an Intersection over Union threshold of 0.5 (mAP@50), and mean Average Precision across multiple IoU thresholds (mAP@50–95). Computational performance is evaluated using frames per second (FPS), reflecting the rate at which the system processes visual input.

In the context of this work, real-time performance is defined as achieving a perception update rate sufficient to support practical decision-making in humanoid robotics. Based on empirical observation and the nature of the evaluated tasks, update rates in the range of 4–8 FPS are sufficient for navigation and object interaction tasks, provided that system latency remains operationally acceptable. Although not explicitly measured, the observed system responsiveness suggests that latency remained within acceptable bounds for task-level robotic control. Tasks such as Marathon, which involve line following and discrete directional decisions, are less sensitive to high frame rates, whereas tasks such as Basketball and Archery benefit from higher temporal resolution.

Compared to traditional geometric vision approaches, the YOLO-based system demonstrated improved robustness under varying lighting conditions, partial occlusion, reflective surfaces, and background clutter. While geometric methods provided higher frame rates, they were more sensitive to environmental variability and object similarity, leading to less stable detections in complex scenes.

Although system latency was identified as an important performance metric, it was not explicitly measured in this study. The perception pipeline includes multiple components: image acquisition, neural network inference, message serialization, and communication via a WebSocket bridge, which collectively contribute to overall latency. While qualitative observations during experiments indicated responsive system behavior, a detailed latency breakdown is left for future work.

## 4. Implementation

The implementation of the YOLO-based vision module on the OP3 humanoid robot represents the transition from system design to real-world deployment. This section details the integration of the trained models into a responsive, practical perception pipeline, the development of ROS-compatible nodes, and the interaction between perception and robot behavior. The implementation emphasizes low-latency processing, modularity, and robustness under constrained computational resources.

### 4.1. Model Deployment

The deployment of the YOLOv9 models follows the training pipeline described in Section 3. Each model is trained using task-specific datasets containing approximately 150 images per class, with annotations generated using Roboflow. Data augmentation techniques, including rotation, exposure adjustment, scaling, and Gaussian blur, are applied to improve robustness under varying environmental conditions.

For inference, a video stream is captured using the ROBOTIS-OP3’s onboard camera. Each frame is processed using a letterbox resizing operation to preserve the original aspect ratio while adapting the image to the required input size of 640 × 640 pixels.

To optimize performance on CPU-only hardware, the trained models are converted into OpenVINO format (see Section 3.3). During inference, bounding boxes are generated based on a confidence threshold, and non-maximum suppression (NMS) is applied to eliminate redundant detections. The resulting bounding boxes are then rescaled to the original image dimensions to ensure accurate spatial alignment with the camera feed.

Visualization is performed using OpenCV, which overlays bounding boxes, class labels, and confidence scores onto the video stream. This functionality supports debugging and validation by allowing inspection of the robot’s perception output.

### 4.2. ROS Node Development

The YOLO-based perception system is implemented using two distinct ROS nodes to address compatibility constraints between ROS Kinetic (Python 2.7) and YOLOv9 dependencies (Python 3.8+). The first node, referred to as the vision node, operates in a Python 3.12 virtual environment and performs object detection using the YOLOv9 model. The second node, referred to as the listener node, operates within the ROS environment and ensures compatibility with other system components.

The vision node is responsible for processing camera input, performing inference using PyTorch and the Ultralytics framework, and generating detection outputs. These outputs include bounding box coordinates, object dimensions, and confidence scores, which are formatted as JSON messages and transmitted via WebSocket. JSON formatting is used to ensure compatibility with WebSocket communication.

For debugging and monitoring, the vision node overlays detection results onto the video stream using and displays the output in real time. Additionally, a cleanup routine is implemented to release camera resources and properly terminate WebSocket connections upon shutdown.

The listener node serves as a bridge between the YOLO inference environment and the ROS ecosystem. It receives JSON-formatted detection messages via WebSocket, parses the data, and republishes it as ROS-compatible messages on designated topics (e.g., /yolo/detections). This enables seamless integration with other ROS nodes.

Error-handling mechanisms are incorporated to manage malformed messages and connection failures. The WebSocket lifecycle is managed through event handlers, ensuring stable communication and allowing for reconnection when necessary. Logging is also implemented to provide visibility into incoming detections and system status.

This dual-node architecture ensures efficient object detection while maintaining compatibility with ROS Kinetic, effectively bridging modern deep learning frameworks with legacy robotic systems.

### 4.3. Pipeline and Behavior Integration

The integration of the vision module into the robotic system is achieved through a structured ROS-based pipeline that enables efficient communication between perception and control components. The system is designed to translate visual detections into meaningful robot actions in real time, particularly within the context of FIRA Hurocup events.

A key challenge in the pipeline design is the incompatibility between Python 3-based YOLO inference and Python 2.7-based ROS Kinetic. This is addressed through a WebSocket-based bridge that facilitates communication between the two environments without interfering with system dependencies. The virtual environment used for YOLO execution is carefully configured to isolate Python 3 libraries and avoid conflicts with ROS.

The vision node transmits detection data via WebSocket as JSON messages, while the listener node decodes these messages and republishes them as ROS topics. This design enables asynchronous, low-latency communication, ensuring responsive robotic perception.

Event-specific nodes subscribe to the detection topics and use bounding box information to guide robot behavior. For example, the robot can adjust its orientation to track detected objects, initiate pick-and-place actions, or perform navigation decisions based on object position and proximity. These behaviors are implemented using custom logic, while actuator control is handled through the Dynamixel SDK.

**Figure 3:**
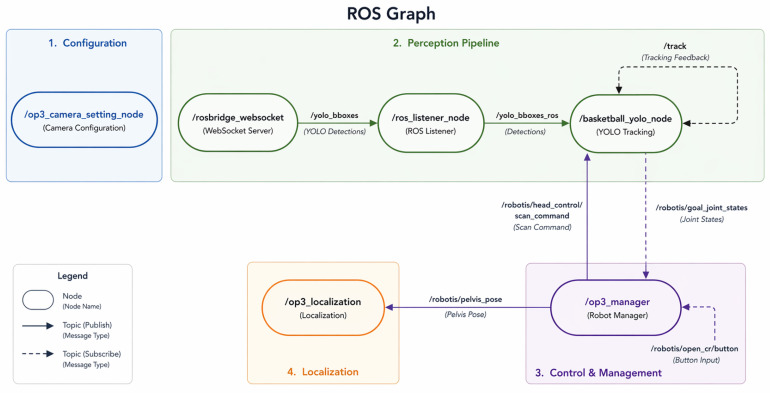
ROS graph illustrating the interaction between perception and behavior modules during the Basketball event. Different colors represent separate functional subsystems, including configuration, perception pipeline, control and management, and localization modules.

The modular publisher–subscriber architecture ensures that each component processes only the information relevant to its function, improving system efficiency and scalability. This design also simplifies integration with existing FIRA event scripts, as the vision module can be incorporated by updating ROS launch files to replace the previous perception system. The complete pipeline during an event is shown in Figure 3.

The integration of the vision module within the ROS ecosystem enables efficient translation of perception outputs into actionable robot behaviors. By decoupling perception from decision-making, the system maintains compatibility with existing ROS components while providing a flexible foundation for future extensions. This modular design supports scalability and adaptability, which are essential for reliable performance in dynamic environments such as FIRA competitions.

### 4.4. Optimization

Optimizing the YOLOv9 inference pipeline is essential to achieve responsive performance on the ROBOTIS-OP3 platform, which is limited to CPU-based computation. Several techniques are employed to reduce latency while maintaining acceptable detection accuracy, including frame skipping, input resizing, model conversion, and quantization.

A simple yet effective optimization is frame skipping, where only every *n*th frame from the camera stream is processed. The parameter *n* is configurable, allowing the system to balance computational load and responsiveness. This approach significantly reduces processing overhead while maintaining sufficient temporal resolution for object tracking and decision-making. Input resizing is also applied to improve efficiency. The raw camera frames are resized to 640 × 640 pixels, which corresponds to the native input resolution of YOLO models. This reduces unnecessary computation on high-resolution images while preserving detection performance. Standardizing the input size further improves preprocessing speed and ensures consistent inference behavior. To enhance CPU inference performance, the trained YOLOv9 models are converted from PyTorch format to ONNX (Open Neural Network Exchange) and subsequently to OpenVINO. ONNX provides a framework-independent representation, while OpenVINO enables hardware-specific optimizations tailored for Intel CPUs. This conversion pipeline reduces computational redundancy and leverages optimized instruction sets, resulting in improved inference speed.

Quantization is applied as a final optimization step to further reduce computational cost. Using PyTorch and ONNX post-training techniques, model weights and activations are converted from 32-bit floating point precision to 8-bit integers. This significantly reduces memory usage and accelerates matrix operations, which dominate inference time. Quantization introduced a minor reduction in detection accuracy, observed qualitatively during testing. However, this effect was not quantitatively measured and is left for future work.

Through the combination of frame skipping, input resizing, model conversion, and quantization, the computational cost of the YOLOv9-based vision system is substantially reduced. These optimizations enable efficient deployment on the OP3 platform, achieving a practical balance between inference speed and detection accuracy for practical robotic applications.

## 5. Results and Discussion

The YOLOv9-based models demonstrate strong detection performance across evaluated tasks, with high precision and recall in controlled conditions. In the Archery task, near-perfect recall is observed, indicating effective target detection. However, this may also reflect potential overfitting due to the relatively small dataset size. While data augmentation was applied, this limitation is acknowledged and discussed further in Section 5.6.

### 5.1. Computational Performance

The system achieves processing speeds of 4–8 FPS on CPU-only hardware. While lower than GPU-based systems, this performance is sufficient for the evaluated robotic tasks. Task-dependent differences are observed. Navigation tasks (Marathon) tolerate lower update rates, while interaction tasks (Basketball, Archery) benefit from higher temporal resolution. Despite these constraints, the system remains functionally effective.

### 5.2. System Responsiveness and Latency Considerations

The proposed architecture introduces additional overhead due to JSON serialization and WebSocket-based communication between the perception module and the ROS control system. However, the impact of this overhead is strongly dependent on the size and frequency of transmitted data.

In this work, only lightweight semantic information (e.g., bounding boxes, class labels, and confidence scores) is transmitted, rather than raw image data. Prior studies have shown that, under such conditions, the overhead introduced by WebSocket-based communication and JSON serialization is relatively small compared to other components in the perception pipeline, such as neural network inference and system scheduling.

For example, recent work on ROS-based perception and communication systems has demonstrated that communication overhead becomes significant primarily when transmitting high-bandwidth data streams (e.g., images or point clouds), while remaining limited for compact semantic messages [48]. Therefore, in the context of this CPU-constrained system, overall latency is expected to be dominated by inference time rather than communication overhead.

Nevertheless, a detailed quantitative latency analysis specific to this implementation remains an important direction for future work.

### 5.3. Comparison with Geometric Vision

The YOLO-based system demonstrates improved robustness compared to traditional geometric methods. While geometric approaches are computationally efficient, they are sensitive to lighting, color ambiguity, and environmental variability. In contrast, the YOLO-based approach provides more reliable detection across varying conditions, at the cost of increased computational demand. This highlights the trade-off between efficiency and robustness.

### 5.4. YOLO Training Results

The training phase of the YOLOv9 models is critical for achieving a balance between detection accuracy and computational efficiency. However, the primary focus of this work is practical deployment on constrained humanoid platforms rather than benchmarking state-of-the-art detector architectures.

A total of nine models are trained, corresponding to three architectures, namely YOLOv9-Tiny (YOLOv9-T), YOLOv9-Small (YOLOv9-S), and YOLOv9-Medium (YOLOv9-M), across three FIRA Hurocup events: Basketball, Archery, and Marathon. All models are initialized with pre-trained MSCOCO weights [44] and trained for 100 epochs with an early stopping patience of five to mitigate overfitting. The training results are summarized in Table 2.

The Basketball models demonstrate consistently strong performance across all metrics. High mAP, precision, and recall values indicate that the models can reliably detect and classify objects with minimal error. This suggests that the visual characteristics of the Basketball event are well represented in the dataset and are relatively easy for the models to learn.

For the Archery task, the models exhibit high mAP@0.5 and perfect recall values but noticeable variation in mAP@0.5–0.95. The consistently high recall values suggest potential overfitting, where the models may be memorizing the training samples rather than generalizing effectively. This indicates limited dataset diversity and suggests that additional data or stronger augmentation may be needed to improve robustness.

**Figure 4:**
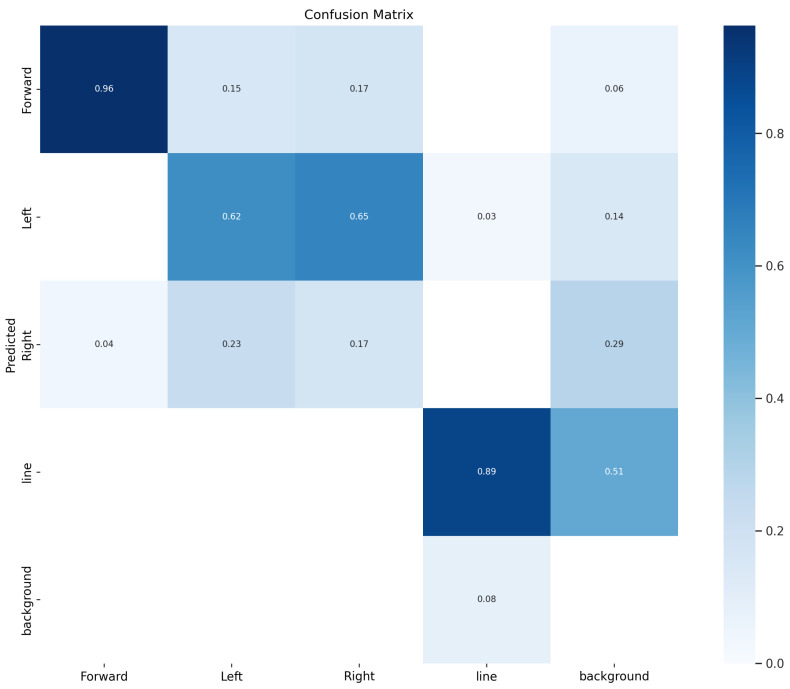
Confusion matrix for the YOLOv9-T marathon model. The matrix shows frequent confusion between left and right arrow markers due to their similar appearance.

The Marathon task yields the lowest overall performance among the three events, with reduced mAP and precision values. Although recall remains relatively high, the lower precision indicates frequent misclassification. As shown in Figure 4, the model struggles to distinguish between left and right arrow markers due to their similar visual appearance.

A key limitation across all models is dataset size and diversity. Due to the computational constraints of the OP3 platform, datasets are limited to approximately 150 images per class, which restricts the models’ ability to generalize. While this constraint supports task-level inference, it also increases the likelihood of overfitting, particularly in tasks with subtle inter-class differences, such as Marathon. Future work could explore transfer learning, synthetic data generation, or expanded augmentation strategies to improve robustness.

Practical robotic performance is a critical requirement for the vision module. To evaluate inference speed, the model architectures are tested under idle conditions without active detections to establish a baseline. All tests are performed with the same optimizations applied.

As shown in Table 3, YOLOv9-T significantly outperforms the larger architectures in terms of frame rate. These results indicate that YOLOv9-T is the only architecture capable of meeting the responsive task-level constraints of the OP3 platform. Consequently, all subsequent experiments are conducted using YOLOv9-T.

### 5.5. Experimental Setup and Procedure

Two types of experiments are conducted to evaluate the performance of the YOLOv9 module in a robotic setting: static tests and dynamic tests. These experiments assess inference speed, operational responsiveness, and detection accuracy under both controlled and operational conditions. In both cases, the YOLO-based module is compared against the geometric approach currently implemented on the robot, which serves as a baseline for evaluating the effectiveness of the proposed vision system.

**Figure 5:**
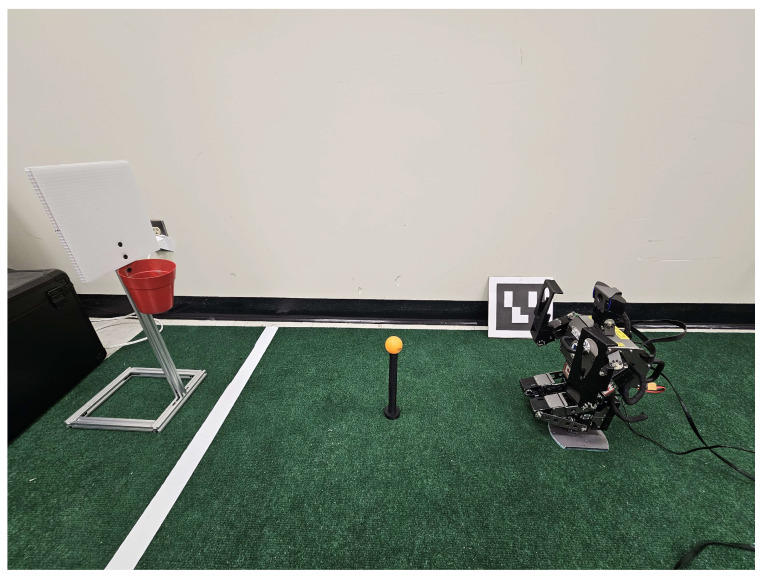
Experimental setup used for both static and dynamic testing in LIMRL. The image shows the setup for the Basketball event on a turf field similar to those used in FIRA competitions.

All experiments are conducted inside the Laurentian Intelligent Mobile Robotics Lab (LIMRL), as shown in Figure 5. The experimental area includes a turf field similar to those used in FIRA competitions, providing a relevant testing environment for the selected tasks.

The static test is designed to isolate the computational performance of the vision system without external variables introduced by robot motion. In this setup, the robot remains stationary while the vision module processes incoming frames. The primary metrics of interest are inference speed and precision, which provide a baseline for assessing computational feasibility and detection reliability.

In contrast, the dynamic test evaluates the vision module under real-world operating conditions by integrating it into the robot’s event-specific scripts. The robot actively interacts with its environment, and the vision system is used to guide decisions in real time. This setup provides insight into how motion, lighting variation, and practical processing demands affect system performance.

The geometric baseline relies on traditional techniques such as color segmentation and predefined geometric rules for object localization and decision-making. Comparing the YOLO-based module against this baseline allows the study to quantify the trade-offs between computational efficiency, detection robustness, and practical usability in robotic applications.

#### 5.5.1. Static Experiments

In the static experiments as shown in Table 4, the YOLOv9 models achieve precision levels comparable to those of the geometric approach across most tasks. In the Marathon event, the YOLO module reports higher precision; however, this result is influenced by confusion between similar arrow marker classes, which inflates detection counts. As a result, the effective true detection rate is likely closer to that of the geometric approach than the reported precision suggests.

In terms of computational performance, the geometric approach consistently achieves higher frame rates, in some cases exceeding YOLO by approximately 50%. Even with the applied optimizations, the YOLO module remains slower than the geometric one. However, the YOLO module provides several practical advantages. Unlike the color-segmentation-based geometric approach, it does not require extensive post-training calibration. In addition, through data augmentation, the YOLO-based module is more robust to environmental variation, such as changes in lighting and background clutter.

**Figure 6:**
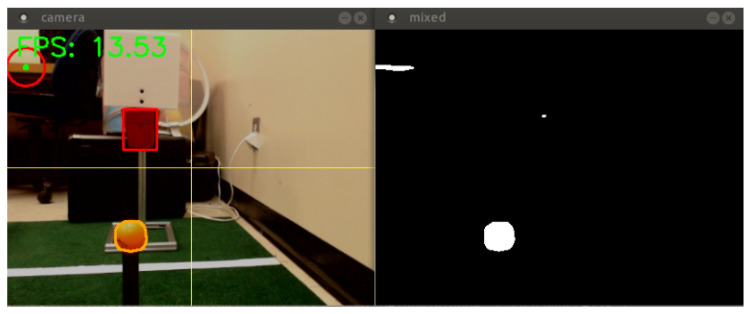
Example of failure in the geometric module during static testing. The red circle and bounding box indicate an incorrect detection caused by lighting variation and background clutter, where background objects are mistakenly identified as part of the ball. The YOLO module is more resilient to this type of visual noise.

As shown in Figure 6, the geometric approach is sensitive to background noise and illumination changes. In contrast, the YOLO module is more robust because the training data includes augmentations such as brightness and rotation adjustments, allowing the detector to generalize more effectively.

#### 5.5.2. Dynamic Experiments

Dynamic experiments introduce real-world constraints such as robot motion, lighting variation, and decision-making. As shown in Table 5, both methods experience lower frame rates than in the static setting, although the relative performance gap remains similar across most tasks.

Performance varies significantly by event. In the Basketball task, the YOLO module outperforms the geometric approach because it is more robust to lighting variability and background clutter. The geometric module relies on contour-based and segmentation-based detection, which becomes less reliable when the appearance of the spherical object changes under different illumination conditions.

In the Archery task, both methods achieve comparable performance. The target has a relatively distinct visual structure and high contrast, which makes it easier for both approaches to detect reliably.

In the Marathon task, the geometric module outperforms the YOLO module. This result reflects the difficulty of distinguishing between visually similar arrow markers using a small dataset. The YOLO module frequently misclassifies marker types, which reduces precision. In this case, the geometric approach benefits from its reliance on predefined shape-based rules.

### 5.6. Discussion

The results of this study demonstrate the feasibility of deploying modern deep learning-based object detection models on resource-constrained humanoid robotic platforms. By leveraging a modular architecture and system-level optimizations, the proposed approach bridges the gap between high-performance computer vision models and practical robotic applications.

A key contribution of this work is the integration of a YOLOv9-based perception module within a dual-environment architecture. This design enables the use of modern deep learning frameworks alongside legacy robotic software systems, addressing a common compatibility challenge in robotics. The use of a WebSocket-based bridge provides flexibility and modularity, allowing the perception and control components to be developed and maintained independently. However, this design choice introduces additional communication overhead, which should be quantitatively evaluated in future work.

The system design is inspired by principles observed in biological vision systems, particularly the modular organization of perception processes. The use of task-specific models reflects the specialization found in biological visual pathways, where different processing streams are responsible for distinct perceptual functions. This modularity enables efficient allocation of computational resources and simplifies model design for specific tasks.

The experimental results highlight important trade-offs between accuracy, robustness, and computational efficiency. While the YOLO-based approach significantly improves detection reliability compared to traditional geometric methods, it operates at lower frame rates due to CPU-only constraints. Nevertheless, the observed performance is sufficient for the evaluated tasks, suggesting that high frame rates are not always necessary for effective robotic perception.

#### Limitations

Several limitations of this study should be acknowledged. First, the dataset used for training is relatively small, which may limit the generalization capability of the models and contribute to overfitting in certain tasks. Although data augmentation techniques were applied, future work should explore larger and more diverse datasets, as well as synthetic data generation, to improve robustness.

Second, system latency was not explicitly measured. While qualitative observations indicate that the system operates responsively, a detailed analysis of end-to-end latency remains necessary to fully evaluate practical performance. The proposed architecture introduces additional overhead due to JSON serialization and WebSocket-based communication. However, as discussed in Section 3.3 and Section 5.2, prior studies suggest that such communication overhead is relatively small when transmitting lightweight semantic data (e.g., bounding boxes and class labels) and that overall latency in perception pipelines is typically dominated by neural network inference and system-level factors. Nevertheless, quantitative latency profiling specific to this implementation is required for a more precise evaluation.

Third, optimization techniques such as model quantization were not quantitatively evaluated. While quantization is widely known to improve inference efficiency on CPU-based systems by reducing computational cost and memory usage, often with only minor reductions in detection accuracy, its impact was not formally measured in this study. In this work, quantization was applied post-training during inference optimization and therefore does not affect the reported mAP values obtained from the trained models. Future work will investigate the trade-offs between accuracy and efficiency introduced by such techniques in the context of humanoid robotic perception.

Finally, while the system was validated on the ROBOTIS-OP3 platform, the proposed architecture is not platform-specific. The modular ROS-based design enables the system to be extended to other humanoid or mobile robotic platforms with similar sensing and computational constraints, supporting broader applicability.

## 6. Conclusions

This paper presents a modular vision system for responsive, practical object detection on resource-constrained humanoid robots. By combining YOLOv9 with a dual-environment architecture, the system enables deployment of modern deep learning models on CPU-only platforms. The results demonstrate that moderate processing rates (4–8 FPS) can still support practical robotic perception while providing improved robustness compared to traditional methods. The study highlights key trade-offs and provides a practical framework for integrating deep learning into constrained robotic systems.

Future work will focus on detailed latency analysis, dataset expansion, and deployment on ROS2-based platforms to further improve performance and scalability. In addition, emerging lightweight and efficiency-optimized object detection models, including recent YOLO variants designed for edge and CPU-based inference, will be explored to further enhance real-time performance under constrained computational conditions.

## Figures and Tables

**Table 1:** Table describing which objects will be included within the dataset, organized by event.

YOLOv9 Training Object Examples		
Model Name	Description	Image
**Basketball**		
Ball	A ping pong ball is used for the kid size, and a larger tennis ball for the adult size.	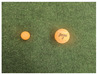
Basket	A bright basketball where the robot must be able to score points on. The kid-sized basket has a diameter of 10 cm, while the adult-sized basket has a diameter of 30 cm.	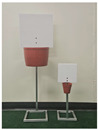
**Archery**		
Target	An archery target with a diameter of 50 cm.	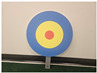
**Marathon**		
Line	A red guiding line, which the robot is tasked with following.	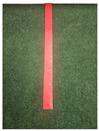
Right Arrow	An arrow whose tip points to the right.	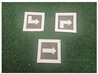
Left Arrow	An arrow whose tip points to the left.	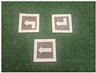
Forward Arrow	An arrow whose tip points straight ahead.	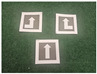

**Table 2:** Results of YOLO model training. All models were trained for 100 epochs with a patience value of 5.

YOLOv9 Training Results				
Model Name	mAP@0.5	mAP@0.5–0.95	Precision	Recall
**Basketball**				
YOLOv9-T	0.99400	0.92536	0.98565	0.98383
YOLOv9-S	0.99500	0.96225	0.98253	0.99809
YOLOv9-M	0.99472	0.95518	0.98590	0.98571
**Archery**				
YOLOv9-T	0.95946	0.92109	0.95934	1.00000
YOLOv9-S	0.98321	0.91407	0.95782	1.00000
YOLOv9-M	0.99393	0.82793	0.95612	1.00000
**Marathon**				
YOLOv9-T	0.75063	0.68905	0.67078	0.89904
YOLOv9-S	0.78545	0.71538	0.61382	1.00000
YOLOv9-M	0.73501	0.61436	0.59230	0.87498

**Table 3:** Results of idle FPS testing. Values are averaged over one minute with no detections to provide a baseline measurement.

Idle Frame Rate Test Results			
Architecture	Basketball	Archery	Marathon
YOLOv9-T	7.99 FPS	8.01 FPS	7.98 FPS
YOLOv9-S	2.75 FPS	2.75 FPS	2.74 FPS
YOLOv9-M	0.86 FPS	0.86 FPS	0.84 FPS

**Table 4:** Results of static testing for the proposed vision module. FPS measures inference speed, and precision represents the percentage of correct detections.

Static Experiment Results		
Method	Speed (FPS)	Precision
**Basketball**		
YOLO Module	5.92	91.60%
Geometric Module	12.99	91.70%
**Archery**		
YOLO Module	6.22	92.24%
Geometric Module	10.64	96.60%
**Marathon**		
YOLO Module	6.12	91.67% *
Geometric Module	8.21	50.8%

* Result affected by confusion between similar arrow marker classes.

**Table 5:** Resultsof dynamic testing for the proposed vision module. FPS measures inference speed, and precision represents the percentage of correct detections.

Dynamic Experiment Results		
Method	Speed (FPS)	Precision
**Basketball**		
YOLO Module	6.68	83.71%
Geometric Module	12.30	50.9%
**Archery**		
YOLO Module	4.41	90.1%
Geometric Module	9.24	93.25%
**Marathon**		
YOLO Module	5.90	39.77%
Geometric Module	8.49	55.5%

## Data Availability

Models supporting reported results can be found at https://doi.org/10.17632/j99hjcryxz.
